# High‐Frequency Stimulated Brillouin Scattering in Suspended Thin‐Film Lithium Niobate Waveguides

**DOI:** 10.1002/nap2.70338

**Published:** 2026-11-13

**Authors:** Lisa‐Sophie Haerteis, Hadi Mahmodi, Yunlong Qiang, Yan Gao, Madeline Hennessey, Peter Thurgood, Jochen Schröder, Arnan Mitchell, Irina V. Kabakova, Michael J. Steel, Andreas Boes

**Affiliations:** ^1^ Institute of Photonics and Advanced Sensing (IPAS) School of Electrical and Mechanical Engineering Adelaide University Adelaide South Australia Australia; ^2^ ARC Centre of Excellence in Optical Microcombs for Breakthrough Science (COMBS) Melbourne Australia; ^3^ School of Mathematical and Physical Sciences University of Technology Sydney Sydney Australia; ^4^ School of Mathematical and Physical Sciences Macquarie University Sydney New South Wales Australia; ^5^ Department of Microtechnology and Nanoscience Chalmers University of Technology Göteborg Sweden; ^6^ Integrated Photonics and Applications Centre (InPAC) School of Engineering RMIT University Melbourne Victoria Australia

## Abstract

Stimulated Brillouin scattering (SBS) enables photonic filtering at gigahertz center frequencies with narrow linewidths at megahertz‐range, making it a key building block for integrated microwave photonics. Accessing radio‐frequency bands beyond 15 GHz with SBS requires high frequency shifts and high gain coefficients, a combination that yet remains a challenge. Here, we show that SBS in suspended Z‐cut thin‐film lithium niobate (TFLN) waveguides satisfies these demands, exhibiting Brillouin frequency shifts exceeding 15 GHz when pumped at telecom wavelengths, nearly twice the highest values previously reported in TFLN. We observe gain coefficients up to 23.8 ${\left(Wm\right)}^{-1}$, among the largest achieved for such high Brillouin frequency shifts in any integrated Brillouin platform to date. For the first time, we apply spontaneous Brillouin microspectroscopy to suspended TFLN waveguides and confirm the high‐frequency acoustic mode observed in our SBS experiments. Our results unlock TFLN as a compelling material platform for scalable microwave photonics, with future applications spanning communication, sensing, and information processing.

## Graphical abstract

Suspended Z‐cut thin‐film lithium niobate waveguides deliver Brillouin frequency shifts above 15 GHz, nearly double previous records in this platform, with gain coefficients up to 23.8 (Wm)⁻¹. Spontaneous Brillouin microspectroscopy confirms the underlying high‐frequency phonon mode, opening thin‐film lithium niobate to microwave photonic filtering well beyond current limits.

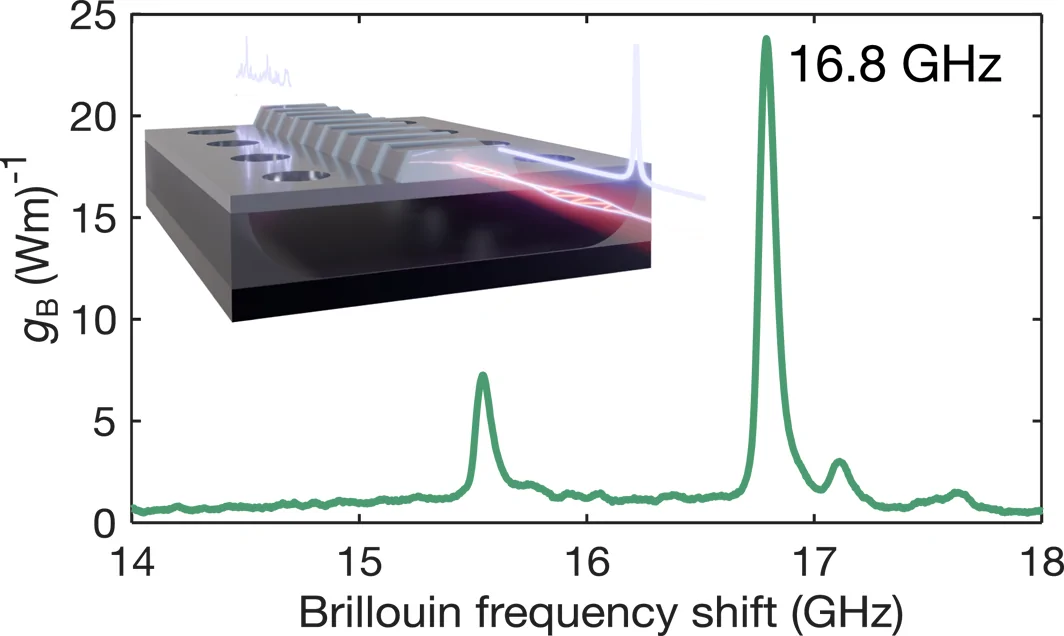

## 1 Introduction

Radio‐frequency (RF) filters are essential to microwave systems, enabling selective signal transmission in crowded spectra. Emerging applications demand fine spectral resolution, broadband tunability beyond 30 GHz, and low size, weight, and power (SWaP), which existing technologies cannot achieve simultaneously [1]. Integrated microwave photonics (IMWP) addresses these challenges by offering low loss, wide frequency agility, and compact implementations [2]. However, realizing fully integrated, chip‐scale microwave photonic processors remains difficult, with suitable material platforms still being a key challenge.

Stimulated Brillouin scattering (SBS) is a key nonlinear effect for IMWP, producing narrow gain resonances (10–100 MHz linewidth) with frequency shifts of 7–11 GHz in typical waveguides. As this lies within microwave bands, SBS enables MHz‐level resolution for GHz‐centered photonic filtering [3]. In this regard, high Brillouin frequency shifts are of importance as they can extend microwave photonic processing into the millimeter‐wave bands, opening direct applications in 5G/6G communications, radar, and satellite links while also relaxing the stringent filtering requirements typically needed to suppress the pump in Brillouin‐based signal processing. Combined with strong Brillouin gain, this is a step toward low‐phase‐noise microwave and millimeter‐wave signal synthesis up to 100 GHz [4].

Longitudinal bulk acoustic modes exhibit higher Brillouin frequency shifts than Rayleigh or shear modes in bulk [5] and inherently propagate at higher acoustic velocities [6]. The same analogy holds true for spatially alike acoustic modes in waveguides. In such context, a quasi‐longitudinal acoustic mode propagates faster than its spatially comparable quasi‐shear equivalent and exhibits a higher Brillouin frequency shift. Confining such modes is a challenge, as efficient acoustic confinement in a waveguide relies on the substrate's acoustic velocity exceeding that of the SBS waveguide material, a requirement that becomes increasingly restrictive at higher Brillouin frequencies. Hence, typical Brillouin material platforms show Brillouin frequency shifts between 7 and 11 GHz at pump wavelengths around 1550 nm, such as chalcogenide [7, 8] and thin‐film lithium niobate (TFLN) [9, 10, 11]. Silicon (Si) [12, 13, 14] and silicon nitride (SiN) [15, 16] platforms have achieved high‐frequency operation; however, Si suffers from two‐photon absorption at high pump powers; SiN, while offering low propagation loss, shows gain coefficients below 1 ${\left(Wm\right)}^{-1}$. Therefore, other material platforms need to be investigated that can simultaneously deliver high Brillouin frequency shifts and large gain coefficients.

TFLN has emerged as a versatile integrated photonic platform, enabled by recent advances in low‐loss waveguide fabrication [17, 18, 19, 20, 21]. Its large electro‐optic and nonlinear coefficients have driven extensive exploration of modulation [22, 23, 24, 25], $\chi^{\left(2\right)}$ [26, 27, 28, 29], and $\chi^{\left(3\right)}$ nonlinear optical processes including four‐wave mixing and supercontinuum generation [30, 31, 32]. Yet another prominent third‐order nonlinear effect [3]—stimulated Brillouin scattering (SBS)—has received comparatively little attention in TFLN, leaving its potential for high‐frequency, high‐gain operation largely unrealized.

Efficient SBS demands simultaneous confinement of optical and acoustic modes, with acoustic confinement proving particularly challenging in TFLN. Two strategies have emerged: surface acoustic wave confinement via total internal reflection, yielding Brillouin shifts of 7–9 GHz [9, 10]; and waveguide suspension, which supports a broader set of acoustic modes and has revealed high gain coefficients in X‐cut [33] and multi‐peak spectra in Z‐cut TFLN [11] at shifts of 8–10 GHz. Despite these advances, understanding of guiding mechanisms in suspended TFLN waveguides remains incomplete, and suppression of acoustic leakage into the substrate could unlock substantially higher Brillouin shifts and gain coefficients.

In this work, we investigate suspended Z‐cut TFLN waveguides and show that suppressing this leakage unlocks a regime previously inaccessible to integrated SBS in TFLN: acoustic modes at 16.8 GHz with a gain coefficient of 23.8 ${\left(Wm\right)}^{-1}$, nearly twice the frequency previously reported in TFLN. This marks TFLN as the only integrated platform that natively combines high‐gain SBS and Brillouin frequencies beyond 15 GHz, while also offering native $\chi^{\left(2\right)}$‐nonlinearity and strong electro‐optic and piezo‐electric responses. We confirm our results by measuring, for the first time, TFLN waveguides using Brillouin microspectroscopy and find good agreement between backward stimulated and spontaneous Brillouin scattering experiments. By extending integrated SBS into the Ku‐band (12–18 GHz) and K‐band (18–26.5 GHz), and toward millimeter‐wave frequencies, our results enable a new class of on‐chip microwave photonic processors for next‐generation communication, sensing, and signal synthesis.

## 2 Brillouin Measurement Configurations

We probe the Brillouin spectrum of suspended TFLN waveguides with two complementary techniques: stimulated Brillouin scattering (SBS) and Brillouin light scattering (BLS) microspectroscopy, as illustrated in Figure 1a,b. In backward SBS, a pump wave and a counter‐propagating Stokes wave detuned downward by several gigahertz are coupled into the waveguide. When the Stokes detuning coincides with the Brillouin shift of a particular elastic mode of the waveguide, that mode is resonantly excited and the Stokes wave experiences efficient amplification.

**FIGURE 1:**
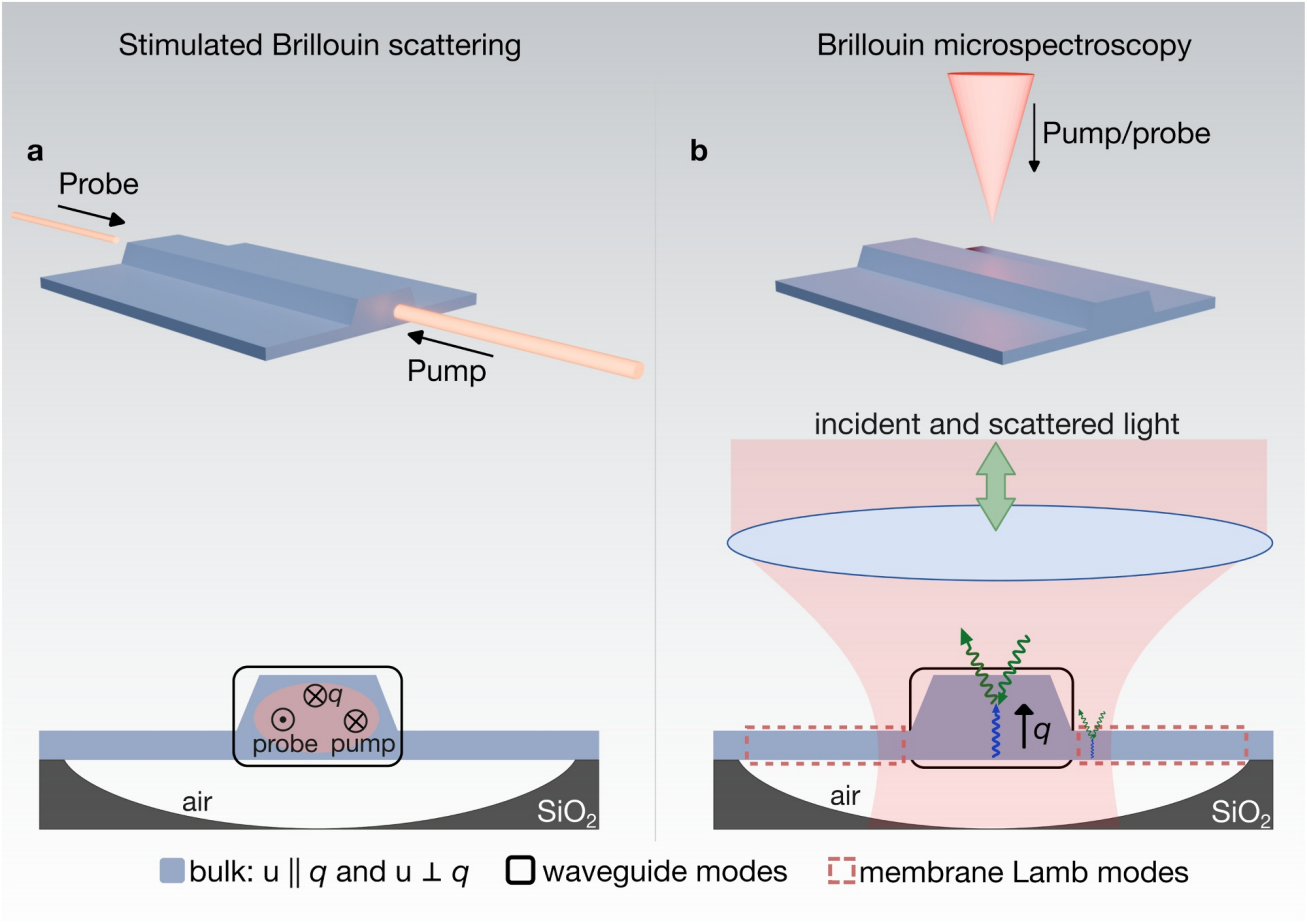
Comparison of stimulated Brillouin scattering and spontaneous Brillouin microspectroscopy performed on suspended waveguides; (a) simplified configuration of backward stimulated Brillouin scattering; cross‐section of the guided geometry. The counter‐propagating pump and probe travel along the waveguide axis, so phase matching fixes the phonon wavevector along that axis with $|q|=4\pi n_{\text{eff}}/\lambda_{\text{SBS}}$. Only modes confined to the ridge (black solid) contribute; the guided optical mode is indicated by the shaded ellipse; (b) simplified configuration of Brillouin microspectroscopy; cross‐section of the microspectroscopy geometry. The focused beam transfers momentum normal to the chip, and the measurement is therefore sensitive to three families: delocalized bulk modes with displacement parallel and perpendicular to q (blue shading), waveguide modes confined to the ridge (solid black), and Lamb modes of the suspended membrane either side of the ridge (red dashed).

In contrast, Brillouin microspectroscopy interrogates *spontaneous* Brillouin scattering. A single incoming laser field (filling the role of the pump in SBS) probes the sample from above. Probe photons can experience inelastic scattering from the bath of thermally excited phonons propagating in the structure (whether as bulk, surface, or confined waveguide modes) and return along the same path to the detection system. The phase‐matching conditions for the particular modes determine the peaks observed at a given illumination wavelength and excitation angle. Since there is no stimulated enhancement, both Stokes and anti‐Stokes features are observed in the spectrum, typically, though not always, with similar strength.

To understand the observed spectra and seek correlations between the stimulated and spontaneous measurements, the phase‐matching conditions are thus critical. As we see below, the final expressions for phase‐matching conditions appear quite different according to the particular process (stimulated or spontaneous), illumination direction and phonon modes involved, and this can lead to the misconception that “different rules” apply to the different setups in a somewhat mysterious fashion. We emphasize that in fact there is a single common set of *potential* phase‐matching conditions that applies to all the configurations; the differences arise according to the different spatial volumes over which the fields interact in each case. Note that since in our experiments, the SBS and BLS illumination are in the near‐IR and visible, respectively, in the following, we distinguish their free‐space wavelengths with subscripts: $\lambda_{\text{SBS}}$ and $\lambda_{\text{BLS}}$.

Taking the down‐shifted Stokes generation for concreteness, the starting phase‐matching conditions are as follows [6, 34]:

(1) $$\omega_{S}=\omega_{p}-\Omega$$

(2) $${\vec{k}}_{S}={\vec{k}}_{p}+\vec{q}\approx -{\vec{k}}_{p},$$

where $\left(\omega_{p},{\vec{k}}_{p}\right)$ and $\left(\omega_{S},{\vec{k}}_{S}\right)$ are the angular frequency and wavenumber of the input pump and output Stokes fields, respectively, and $\left(\Omega ,\vec{q}\right)$ are the corresponding quantities for the elastic field. Critically, the wavevector condition is a vector‐valued relation, and not every component of the condition is realized in all coupling geometries.

To understand this, recall that the phase‐matching conditions essentially arise from complex exponential factors of the form $\exp\left(-i\left(\omega_{S}+\Omega -\omega_{p}\right)t\right)$ and $\exp\left(i\left({\vec{k}}_{S}+\vec{q}-{\vec{k}}_{p}\right)\cdot \vec{r}\right)$ sitting inside the relevant nonlinear overlap integral. If these terms (and any accompanying slowly varying envelopes) are integrated over comparatively large regions in time or space, they effectively collapse to delta functions $\delta \left(\omega_{S}+\Omega -\omega_{p}\right)$ and $\delta^{3}\left({\vec{k}}_{S}+\vec{q}-{\vec{k}}_{p}\right)$ imposing the above phase‐matching conditions. With the many‐nanosecond timescales in Brillouin interactions, frequency condition (1) in principle holds to a high degree but is broadened by the acoustic dissipation.

The wavevector conditions (2) also apply in full for both the waveguide SBS process and for spontaneous Brillouin scattering by *bulk* elastic modes. In both cases, the optical and elastic fields overlap over distances of many wavelengths along the optical path. For the SBS case, the fields are the optical and elastic waveguide modes propagating along the surface with wavevectors ${\vec{k}}_{i},\vec{q}\|\hat{x}$ and the resulting phase‐matching condition is

(3) $$q_{\text{SBS}}\approx 2k_{p}=\frac{4\pi n_{\text{eff}}}{\lambda_{\text{SBS}}},$$

where $n_{\text{eff}}$ is the effective index of the waveguide mode involved. For the bulk mode spontaneous scattering, the fields are plane wave states collinear with the refracted light paths in the bulk volume as determined by Snell's law. For the spontaneous scattering of bulk elastic modes, the incoming probe light is refracted into the body of the sample and the phonon wavevector must reverse the direction of light propagation: the full vector condition again applies so that [35]

(4) $$q_{\text{bulk}}=|{\vec{q}}_{\text{bulk}}|=|2{\vec{k}}_{p}|=\frac{4\pi n}{\lambda_{\text{BLS}}}$$

where ${\vec{k}}_{p}$ is the wavevector of the refracted probe light and n is the refractive index of the sample, with the result exactly paralleling the SBS result in Equation (3).

However, for spontaneous scattering by surface or guided elastic modes, although the interaction is extended along the surface in the $\hat{x}$ direction, vertically it is confined to a region of less than a micron below the surface and this is insufficient to activate the phase‐matching condition along the vertical $\left(\hat{z}\right)$ component of Equation (2). Consequently, only the surface $\hat{x}$ component of Equation (2) is enforced. The tangential component of the optical field $k_{x}=k_{\text{BLS}}\;\sin\;\theta$ is conserved upon refraction into the crystal and the phase‐matching condition becomes [35]

(5) $$q_{\text{surf}}=2k_{x}=2k_{\text{BLS}}\;\sin\;\theta =\frac{4\pi \;\sin\;\theta }{\lambda_{\text{BLS}}},$$

where θ is the angle of incidence of the probe beam from the surface normal. We note unlike the previous two conditions, this expression is independent of the refractive/effective index which can be surprising at first.

A conceptual schematic of an elastic mode dispersion diagram, with indicative wavevectors $q_{\text{SBS}},q_{\text{bulk}}$, and $q_{\text{surf}}$, is depicted in Figure 2. Here, we show dispersion curves for bulk, guided, and membrane Lamb acoustic modes, as bulk and Lamb modes can be measured with BLS microscopy (as seen in Figure 1). A consequence for the Brillouin shifts measured by the two experimental methods follows directly: the phase‐matched wavevectors probe distinct regions of dispersion which is why the Brillouin frequencies measured in the two experiments differ and do not necessarily relate linearly.

**FIGURE 2:**
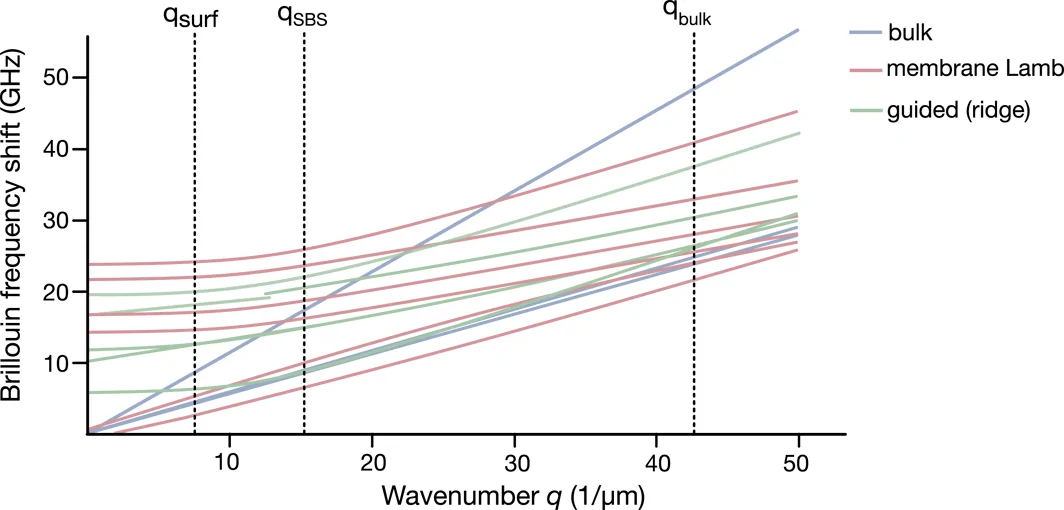
Conceptual schematic of the elastic mode dispersion diagram, indicating the expected regions of phase‐matched wavevectors for different modes and illuminations.

## 3 Waveguide Fabrication

Waveguides are fabricated from a commercially available Z‐cut TFLN 4‐inch wafer (NANOLN) using EBL and a dry etching method, starting from a 520‐nm thick TFLN layer. To form waveguides, the TFLN layer is dry‐etched down to a slab of 200 nm thickness, which results in trapezoidal‐shaped waveguides at a sidewall angle of 70°. The waveguides are then suspended using a wet etching method where the chip is immersed in a 49% HF solution for 5 minutes. The suspended waveguide is eventually created as the 49% HF solution removes the underlying silica through the fully etched holes. Top‐down views illustrating the waveguide and the holes enabling underetching are shown in Figure 3. The detailed fabrication process are described in refs. [11, 36, 37].

**FIGURE 3:**
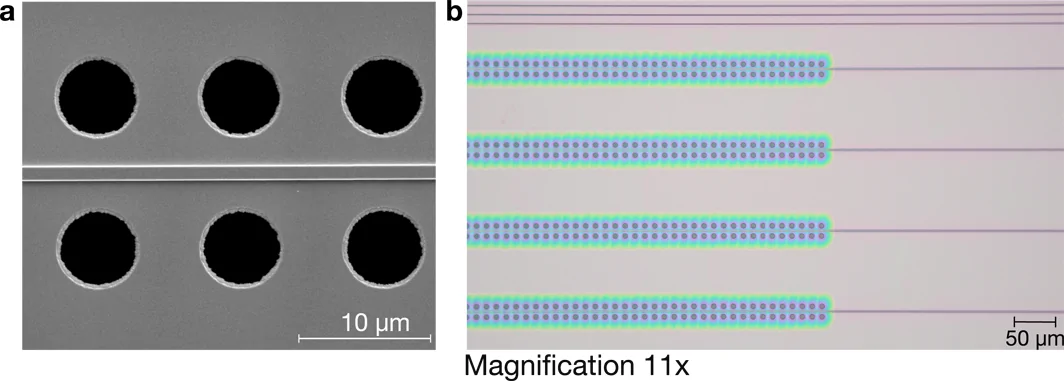
Representative SEM and microscopic images of suspended TFLN waveguides; (a) SEM top‐view of a suspended waveguide with the fully etched holes next to the waveguide which allow for the suspension; (b) microscopic top‐view image of suspended TFLN waveguides.

## 4 Backward Stimulated Brillouin Scattering

### 4.1 Numerical Calculation

We begin with a numerical study of the acoustic modes that mediate efficient SBS interactions using COMSOL [9], to first solve for optical and acoustic modes and then evaluate Brillouin gains from overlap integrals of the relevant modes. Notably, the COMSOL model considers the piezoelectric behavior of lithium niobate. The material properties and model can be found in ref. [9].

Acoustic confinement relies on a substantially lower acoustic velocity in the core material when compared to the substrate material to prevent acoustic leakage and enable an efficient SBS process. As we entirely remove the underlying substrate and create suspended TFLN waveguides, we expect to guide acoustic modes with velocities exceeding those in the typical case of a TFLN‐on‐$SiO_{2}$ waveguide. This might allow us to not only trap shear but also longitudinal acoustic modes, which typically exhibit significantly higher velocities than shear modes, as outlined in ref. [11].

To investigate confinable acoustic modes, we use the suspended structure for simulation in COMSOL, as depicted in Figure 4a. It consists of a rib waveguide of trapezoidal profile suspended in vacuum, supported by a membrane of sufficient thickness to support the waveguide. We take a waveguide thickness t=520 nm, a membrane thickness of $h_{m}=200$ nm, and a waveguide top width of w=1500 nm. The pump wavelength is set to 1550 nm.

**FIGURE 4:**
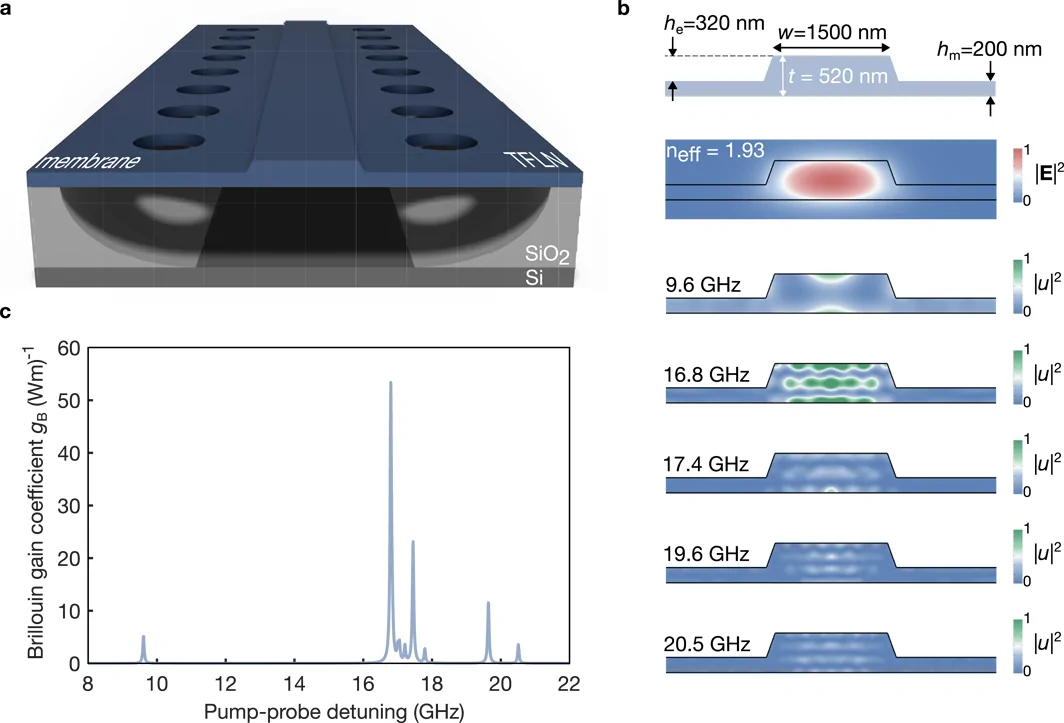
Numerical study of suspended Z‐cut TFLN waveguide for backward stimulated Brillouin scattering; (a) suspended TFLN waveguide; (b) dimensions of the TFLN waveguide cross‐section. Normalized electric field of the quasi‐TE optical mode and normalized displacement field of the acoustic modes; (c) numerically calculated Brillouin gain spectrum of the suspended TFLN waveguide, predicting several higher frequency modes with a high Brillouin gain coefficient.

The Brillouin gain spectrum is depicted in Figure 4c, indicating Brillouin frequency shifts falling between 8 and 20 GHz, crossing at least two microwave bands (X‐band and Ku‐band). The spectrum exhibits the largest response at 16.8 GHz, with a larger gain coefficient than the mode at a frequency of 9.6 GHz. Notably, Brillouin shifts higher than 10 GHz have not been predicted nor measured in TFLN before [9, 10, 11, 38]. Figure 4b illustrates the fundamental optical mode, showing tight confinement with an effective index of $n_{\text{eff}}=1.93$ for a quasi‐TE mode, along with the displacement fields of the acoustic modes corresponding to the five largest gain peaks. The modes across the frequency range are fundamentally different. The mechanical polarization fractions extracted from the simulation show the difference between the phonon modes at 9.6 and 16.8 GHz: the first is strongly y‐polarized with x‐, y‐, and z‐polarization fractions $f_{x}=0.11,f_{y}=0.79,f_{z}=0.09$, respectively, (a shear vertical mode, i.e., essentially a horizontally confined Lamb mode coupling surface excitations at the upper and lower surface). The 16.8 GHz mode is predominantly z‐polarized with polarization fractions $f_{x}=0.05,f_{y}=0.12,f_{z}=0.82$ (a longitudinal mode). Note that our measure of the polarization fractions is defined in the Materials and Methods Section.

As the acoustic frequency relies on the pump wavelength, the acoustic velocities additionally give insights into the nature of the phonon mode. The resonances at 9.6 and 16.8 GHz have acoustic velocities of 3872 m/s and 6772 m/s, respectively, consistent with the resonance at 16.8 GHz having a strong longitudinal character. In comparison, the shear and longitudinal acoustic velocities in silica $\left(SiO_{2}\right)$ are ≈ 3700 m/s and ≈ 6000 m/s, respectively. Particularly, the numerically observed acoustic modes at 16.8 GHz would hence leak into the $SiO_{2}$ substrate for a non‐suspended waveguide, which would result in a suppressed SBS response. The Brillouin gain spectrum of a non‐suspended waveguide in Figure 4c (with the same dimensions as shown in Figure 4b) supports this by exhibiting a low‐amplitude peak at 9.8 GHz and a flat response beyond that. This indicates the necessity of waveguide suspension in order to confine high‐frequency, high‐velocity acoustic modes in TFLN waveguides.

### 4.2 Experimental Results

We investigate stimulated Brillouin scattering measuring optically induced acoustic modes in the suspended Z‐cut TFLN waveguide using a pump‐probe measurement technique [15]. Details on the experiment can be found in the Materials and Methods Section.

Figure 5a depicts the measured Brillouin gain spectrum of the suspended waveguide for pump‐probe detuning between 8 and 22 GHz. The spectrum shows four SBS gain peaks from acoustic modes at frequencies between 8 and 10 GHz. In addition, the spectrum shows the first experimental evidence of a set of high‐frequency acoustic modes in TFLN, with peaks at 15.5 GHz, 16.8 and 20.2 GHz, with the strongest response at 16.8 GHz. Estimating the Brillouin gain coefficient according to the authors in ref. [15] further reveals a Brillouin gain coefficient of 23.8 ${\left(Wm\right)}^{-1}$ at a Brillouin shift of 16.8 GHz. We fit the spectral response using a Lorentzian line shape via nonlinear least‐square optimization, yielding a full width half maximum of 91.3 MHz for the 16.8 GHz acoustic mode (Q≈184) and 93.5 MHz for the 15.5 GHz mode (Q≈166).

**FIGURE 5:**
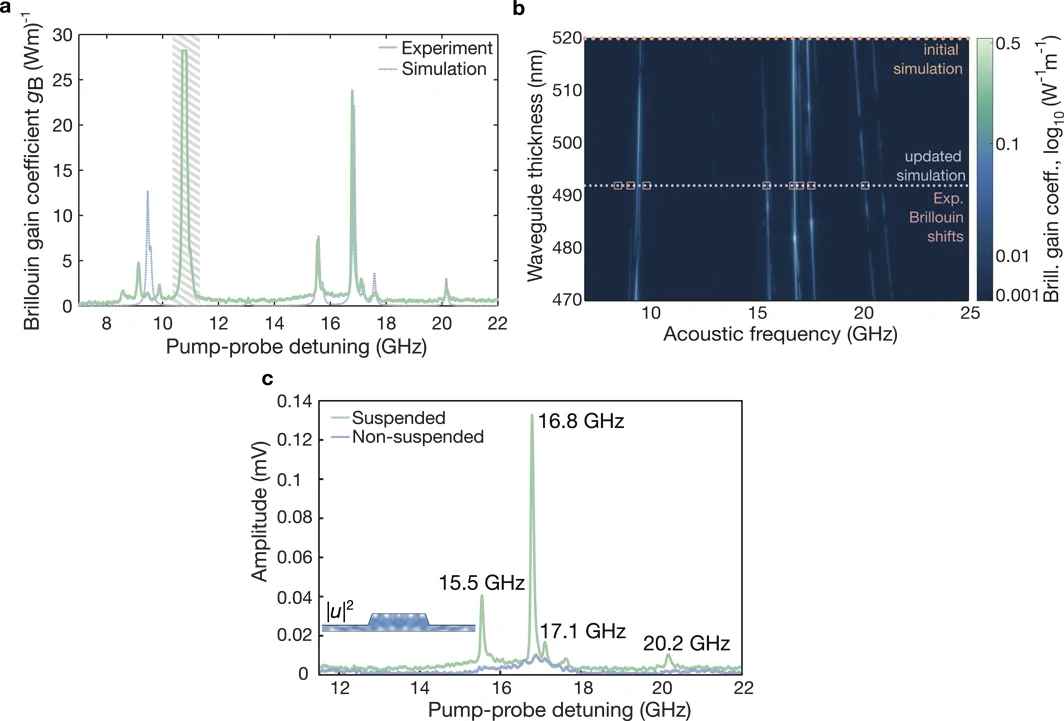
Backward‐stimulated Brillouin scattering in suspended thin‐film lithium niobate waveguides; (a) measured Brillouin gain spectrum from 8 to 22 GHz for waveguide width w=1500 nm, waveguide thickness t=520 nm, and membrane thickness $h_{m}=200$ nm. The large peak at 10.8 GHz originates from fiber SBS; (b) simulated Brillouin spectra for a sweep of waveguide thickness t and membrane thickness $h_{m}$ with a fixed etching depth of $h_{e}=320$ nm and waveguide width of w=1500 nm. Color scale is shown on a logarithmic $\left(\log_{10}\right)$ axis to highlight weaker acoustic modes alongside the dominant contribution; the red squares indicate the experimental Brillouin shifts and show the best overlap with a waveguide and membrane thickness of t=492 nm and $h_{m}=172$ nm, respectively; (c) Brillouin gain spectrum comparison of suspended and non‐suspended waveguides.

The simulated and experimental Brillouin gain spectra in Figure 4c and 5a, respectively, both display a dominant Brillouin resonance at 16.8 GHz, confirming that the simulation captures the principal acoustic mode in the suspended TFLN waveguide with excellent accuracy. Beyond this main feature, a closer inspection of the lower‐amplitude peaks reveals subtle differences between simulation and the experiment, which we discuss in the following. Mainly, the experimental acoustic mode at 15.5 GHz is absent from our initial numerical prediction (see Figure 4c), whereas the simulation shows a larger peak at 17.4 GHz that is significantly lower in amplitude in the experimental results. The difference in simulation and measured results is likely caused by the modeled waveguide geometry which may differ slightly from the fabricated structure. The underetching procedure attacks the ‐Z‐face of TFLN (which is the underside of the waveguide), but at a much lower etching rate (approx. 10 nm/min [39]) compared to silica, and so reduces both the waveguide and membrane thickness. Thus, we performed a numerical scan, sweeping the waveguide thickness t and membrane thickness $h_{m}$ with a fixed waveguide width of w=1500 nm, a sidewall angle of 70°, and a fixed etching depth of $h_{e}=320$ nm (difference between waveguide and membrane, meaning $h_{m}=t-320$ nm) thickness (for simplicity, we assume an identical reduction of the waveguide and membrane thicknesses).

The evolution of the calculated Brillouin gain spectra with reducing waveguide and membrane thickness t and $h_{m}$, respectively, is shown in Figure 5b. Tracing individual resonances in the image, it is apparent that slight changes in the geometry of order 5 nm have a significant impact on the numerical acousto‐optic overlap strength for some resonances. The gain coefficient of the resonance close to 16 GHz is notably changing with thickness, and for t>510 nm, the strength of the resonance might be too weak to capture in an experiment. This explains why the resonance, although clearly visible in our experiment, is absent from the initial simulation (highlighted in Figure 5b) at t=520 nm. Notably, the strongest resonance at 16.8 GHz is much less sensitive to geometry deviations. We find the Brillouin gain spectrum closest to our experimental results at a waveguide thickness of t=492 nm (the membrane thickness is $h_{m}=172$ nm) highlighted in Figure 5b and separately shown in Figure 5a. It indicates good agreement with the experiment, particularly for the resonances at 15.5 and 16.8 GHz. The 15.58 GHz resonance, absent from our initial simulation, has an acoustic velocity of 6348 m/s, and the polarization fractions of $f_{x}=0.26$, $f_{y}=0.22$ and $f_{z}=0.52$ indicate that this mode is a hybrid of shear and longitudinal modes. Its displacement field is depicted in Figure 5c. Although the Brillouin gain spectra of simulation and experiment are in good agreement above 10 GHz, discrepancy is evident in the lower Brillouin frequency shift regime. The simulation predicts confinement of two acoustic modes between 8 and 10 GHz as indicated by the shouldered peak, where the peaks of two acoustic modes overlap. The experiment, however, shows confinement of 4 acoustic modes with lower Brillouin gain coefficients between 8 and 10 GHz.

We would like to note that, the HF solution etches TFLN at an etching rate of 10 nm/min and would theoretically result into a TFLN thickness of 470 nm. However, according to the agreement between simulation and experiment, it appears that the thickness of the TFLN layer is closer to t=492 nm. This discrepancy might stem from the fact that the TFLN layer is not uniformly etched. The HF solution attacks the $SiO_{2}$‐layer isotropically from the fully etched holes—and only affects TFLN once it is exposed. The exposure of TFLN occurs gradually and will affect the area underneath the waveguide core area the least, which is where the confinement of optical and acoustic modes takes place.

In Figure 5c, we additionally measure the Brillouin spectrum of a non‐suspended waveguide with w=1500 nm, t=520 nm and $h_{m}=200$ nm and compare it to the suspended waveguide, focusing on the pump‐probe detuning area between 12 and 22 GHz. The marginal signal elevation in case of the non‐suspended waveguide supports our theory that acoustic modes with velocities larger than those of the underlying $SiO_{2}$‐substrate will leak and the SBS response suppressed, which is in a good agreement with our numerical predictions in Figure 4c.

Our experiments show evidence of high‐frequency Brillouin shifts that are nearly twice the frequency of previously reported confined modes [9, 10, 33, 40]. Additionally, the Brillouin gain coefficients for shifts higher than 10 GHz are significantly larger compared to those between 8 and 10 GHz. The Brillouin gain spectra comparison acquired from non‐suspended and non‐suspended waveguides confirms the necessity of waveguide suspension to confine the high‐frequency phonon modes.

## 5 Spontaneous Brillouin Scattering via Brillouin Microspectroscopy

We now turn to the insights that can be obtained from the study of *thermally*‐excited acoustic modes via spontaneous Brillouin scattering experiments. We use Brillouin microspectroscopy [41, 42, 43] to independently verify the 16.8 GHz mode and characterize the acoustic modes in suspended TFLN waveguides. This method scatters light from the intrinsic thermally excited acoustic modes in the suspended TFLN waveguide and surrounding TFLN membrane, using an excitation laser at wavelength λ=660 nm. The Brillouin scattering efficiency is proportional to $\lambda^{-4}$, so the scattering signal is significantly weaker at long wavelengths [44], which is why 660 nm is generally preferred over 1550 nm. The backscattered Brillouin shifted light is detected by a 6‐path tandem Fabry–Pérot interferometer. Details on the experimental setup can be found in the Methods section. We note that although the 16.8 GHz acoustic mode arises from stimulated Brillouin scattering pumped at 1550 nm, BLS microscopy excites spontaneous Brillouin scattering pumped at 660 nm. The acoustic frequencies observed in the Brillouin microspectroscopy experiment therefore inherently differ from the SBS experiment, owing to the large difference in pump frequencies.

The TFLN waveguide sample is tilted by θ=25° to capture waveguide modes with a component oriented in plane with the waveguide. We chose the tilting angles of 20°, 25°, and 30° deliberately as tilting angles below 20° result in the back‐reflection of the pump, masking the signal of interest which is the Brillouin scattered signal that is of low amplitude. Beyond 30°, the backscattered light falls out of the collection aperture of the objective lens (NA = 0.42). Figure 6a shows the acquired spectrum at a tilting angle of 25°, where we extract the Brillouin shifts of thermally excited modes at acoustic frequencies of 12.1, 17.4, 21.8, 24.3, 28.4, 41.2, and 48.8 GHz.

**FIGURE 6:**
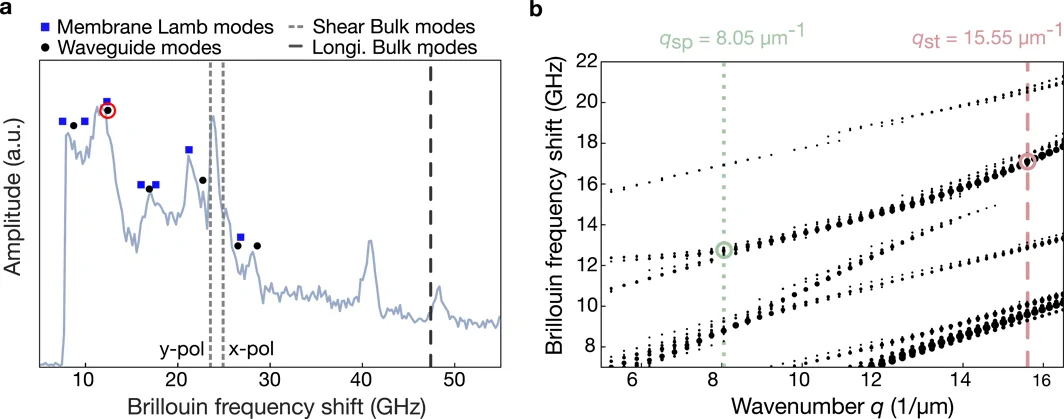
Spontaneous Brillouin light scattering spectrum of a TFLN waveguide measured by Brillouin microspectroscopy; (a) the measured data compared to numerically calculated Brillouin shifts of bulk, waveguide, and membrane Lamb modes at θ=25°; (b) calculated dispersion map of waveguide modes confined in the suspended TFLN waveguide structure.

We first identify each of the modes extracted from the Brillouin microspectroscopy experiment and start by comparing the probing laser beam mode field diameter (MFD) of 2 μm with the waveguide width of 1.5 μm. Because the MFD exceeds the waveguide width in spontaneous Brillouin measurements, we may collect light scattered by phonons not only localized to the TFLN waveguide but also those traveling in the surrounding membrane. Furthermore, since we probe the waveguide from outside, both bulk and guided modes may be expected to be found in the phonon spectrum. For the identification purposes, we simulate bulk, waveguide, and Lamb modes in TFLN with an excitation wavelength of 660 nm in which we also consider the sample tilting angle of θ=25° to fully represent the scattering geometry of the experiment. The simulation results are depicted in Figure 6a, indicated by dots for the waveguide (guided) modes, squares for the membrane modes, and dashed lines for the bulk shear and longitudinal modes. Overall, simulation and experimental results show close agreement and enable the identification of individual acoustic modes: (i) shear and longitudinal bulk modes appear at 24.3 and 48.8 GHz; (ii) waveguide modes appear at 12.1 GHz, 17.4 GHz, and 28.4 GHz; and (iii) a membrane Lamb mode appears at 21.8 GHz. One assignment requires clarification: a slab Lamb mode is in close vicinity to the thermal phonon mode at 12.1 GHz (as can be seen in Figure 6a). However, the acousto‐optic overlap would not lead to a significant response in the spontaneous Brillouin spectrum; hence, we assume the mode at 12.1 GHz to be a waveguide mode.

We now assign the dominant acoustic mode at 16.8 GHz encountered in the stimulated Brillouin scattering experiment (with excitation wavelength $\lambda_{\text{st}}=1550$ nm) to its corresponding thermally excited phonon mode in the spontaneous experiment (with excitation wavelength $\lambda_{\text{sp}}=660$ nm). Because the two experiments probe different wavevectors, we cannot compare their acoustic frequencies directly, but need a dispersion map to translate between them. For this purpose, we calculate the in‐plane wavevector (parallel to the surface of the chip) $q_{\|}$ for the stimulated and spontaneous Brillouin scattering cases, using Equations (3) and (5)

(6) $$q_{\|,SBS}=\frac{4\pi n_{\text{eff}}\left(\lambda_{\text{SBS}}\right)}{\lambda_{\text{SBS}}}=15.6\;\mu m^{-1}$$

(7) $$q_{\|,surf}=\frac{4\pi \;\sin\left(\theta \right)}{\lambda_{\text{BLS}}}=8.05\;\mu m^{-1}\;for\;\theta =25{}^{\circ},$$

with θ the tilting angle used to acquire the spontaneous Brillouin spectrum.

The difference in wavenumbers is important in comparing the acoustic frequencies of phonon modes from the two experimental approaches. To find the correct acoustic frequency for the spontaneous phonon mode, we calculate a dispersion map shown in Figure 6b, which shows simulated waveguide modes over the (general) wavenumber $q_{\|}$. The dispersion map is calculated using the open‐source finite‐element tool NumBAT [45, 46] in a suspended Z‐cut TFLN structure, where the geometry is a trapezoidal waveguide with width 1500 nm and height 320 nm (with a 70° waveguide sidewall angle for the trapezoid), sitting on a slab/membrane of the same material with width 2750 nm and height 200 nm. For the simulation, we assume that this structure is surrounded by vacuum (with enough distance to prevent interaction with simulation boundaries). The simulation is essentially running as a stimulated Brillouin scattering system, where light of a specific wavenumber q enters the cross‐section of the structure through edge coupling, as we are particularly interested in the in‐plane wavevector $q_{\|}$. To obtain the dispersion map, we sweep the wavenumber q and calculate the acoustic modes generated within the structure for each wavenumber. This allows us to find the frequencies of the modes which appear at each wavenumber. We then determine a dispersion map, by tracking the frequencies of the modes which emerge (not every single mode is displayed, but only particular modes with the highest gain in our frequency region of interest).

We locate our mode of interest in the dispersion map through its acoustic frequency at 16.8 GHz and the calculated wavenumber $q_{\|,SBS}=15.6\;{\mu m}^{-1}$ (red circle in Figure 6b). We then follow the same waveguide mode until we hit the wavenumber of $q_{\|,surf}=8.05\;{\mu m}^{-1}$ (green circle in Figure 6b), from which we can determine the respective acoustic frequency that is expected in the spontaneous Brillouin scattering experiment. The extracted Brillouin frequency of 12.4 GHz (according to the green circle in Figure 6b) is in close agreement with the experimental (spontaneous) Brillouin frequency of 12.1 GHz (Figure 6a), so we consider this acoustic mode as a confirmation of the high‐frequency mode at 16.8 GHz from the stimulated Brillouin scattering experiment.

In this section, we confirm the acoustic mode at 16.8 GHz from the stimulated Brillouin scattering experiment, supporting our observations of high‐frequency phonon modes by stimulated Brillouin scattering. Beyond this verification, these experiments mark the first experimental realization of Brillouin microspectroscopy with waveguides using suspended thin‐film lithium niobate waveguides [41, 47]. We also note that Brillouin microspectroscopy can probe acoustic modes supported by the full system consisting of waveguide ridge and surrounding membrane. This is complementary to stimulated Brillouin scattering experiments which are sensitive only to acoustic modes with strong overlap with the guided optical mode.

## 6 Discussion

The field of integrated stimulated Brillouin scattering has consistently faced a trade‐off between the Brillouin gain coefficient and frequency shift. In Figure 7, we compare state‐of‐the‐art platforms, revealing two distinct regimes: high gain at low‐to‐moderate Brillouin frequency shift and moderate‐to‐low gain at higher frequencies.

**FIGURE 7:**
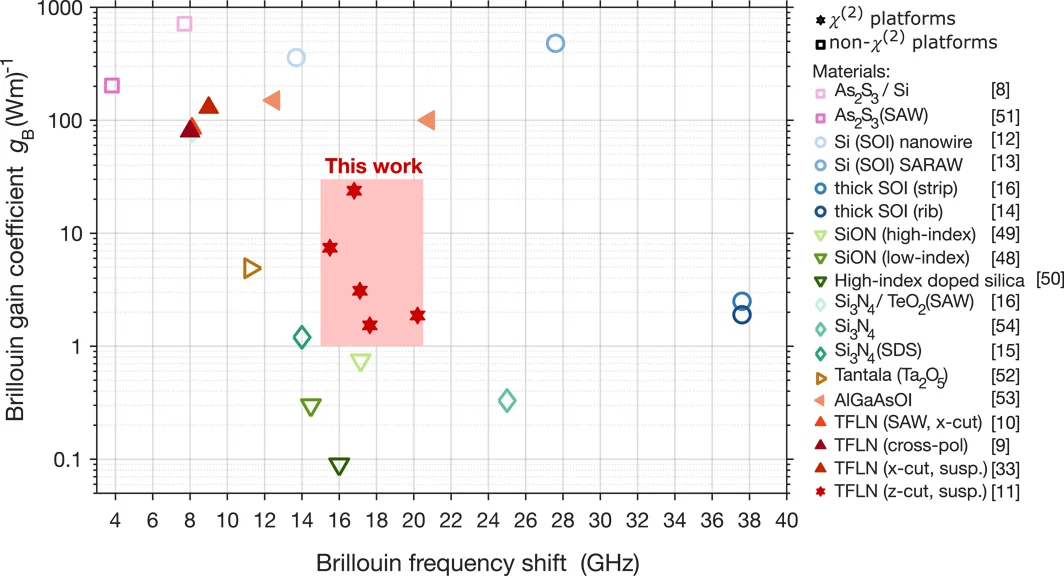
State of the art of backward stimulated Brillouin scattering in integrated material platforms [8, 9, 10, 11, 12, 13, 14, 15, 16, 33, 48, 49, 50, 51, 52, 53]. Solid markers indicate $\chi^{\left(2\right)}$‐material platforms, whereas hollow markers indicate material platforms without $\chi^{\left(2\right)}$‐nonlinearity.

The highest reported gain coefficients and net gains are achieved by chalcogenide glass (${\text{As}}_{2}S_{3}$) [8, 51] and silicon (Si) [12, 13] platforms. ${\text{As}}_{2}S_{3}$ is fixed around 7.7 GHz by the material's acoustic velocity, whereas silicon is fundamentally constrained at telecom wavelengths by two‐photon absorption and the resulting free‐carrier absorption, which might cap the usable pump power. High frequency shifts are achieved by silicon nitride [15, 16, 54] and silicon oxynitride [48, 49], which benefit from ultra‐low optical propagation loss and CMOS compatibility and reach shifts as high as 17 GHz, but the specific photoelastic response of these materials results in gain coefficients below 1.2 ${\left(Wm\right)}^{-1}$, making net gain difficult to achieve in practice. Native $\chi^{\left(2\right)}$‐nonlinearity, required for multifunctional integration with electro‐optic modulation, frequency conversion, and comb generation, is offered only by AlGaAs‐on‐insulator [53] and TFLN [9, 10]: AlGaAs may suffer from two‐photon absorption at telecom wavelengths, whereas existing TFLN demonstrations are driven by surface acoustic waves that confine operation to frequencies below approximately 9 GHz. The suspended anti‐resonant acoustic waveguide (SARAW) geometry in silicon [13] is a notable outlier, extending the Brillouin frequency shift to a record of 27.6 GHz with a gain coefficient of 480 ${\left(Wm\right)}^{-1}$, coming with power scalability limitations imposed by two‐photon absorption, and SARAW geometries are highly selective, being optimized for a specific acoustic mode.

Figure 7 shows that, apart from the SARAW outlier and AlGaAs, there is a gap for materials offering Brillouin shifts beyond 15 GHz with Brillouin gain coefficients larger than 2.5 ${\left(Wm\right)}^{-1}$. The present work (hexagon markers in red) addresses this gap, demonstrating high Brillouin frequency shifts >15 GHz with $g_{B}>1$ ${\left(Wm\right)}^{-1}$ and particularly a Brillouin gain coefficient of 23.8 ${\left(Wm\right)}^{-1}$ at a Brillouin shift of 16.8 GHz. This Brillouin response is only accessible through the removal of the substrate to enable confinement of phonons with acoustic velocities exceeding those of the substrate material. Crucially, we do so on a platform that natively supports $\chi^{\left(2\right)}$‐nonlinearity and exhibits a large electro‐optic and piezo‐electric response, meaning that high‐frequency, high‐gain SBS can now be co‐integrated with electro‐optic modulation, frequency comb generation, and second‐harmonic generation on a single chip.

Further improvements are required to achieve high net gains in suspended TFLN waveguides required for system‐level applications. With the goal of maximizing the SBS gain, we consider the relation $G=e^{g_{B}P_{P}L_{\text{eff}}}$, showing the exponential dependence of the gain on the Brillouin gain coefficient $g_{B}$, pump power $P_{P}$, and effective interaction length $L_{\text{eff}}$. Facet losses currently exceed 5 dB per facet and limit the on‐chip pump power, and reducing these to a theoretical minimum of ∼1 dB would directly increase the on‐chip pump power. With regard to the gain coefficient, maximization through improving fabrication (including the underetching technique) and carefully tailoring the waveguide geometry can improve the overall SBS gain as shown in Figure 4b, for example, buffered oxide etchant (BOE) might be used to selectively etch the $SiO_{2}$‐layer without etching the ‐Z LN film, improving the accuracy of waveguide geometry predictions. Increasing the suspended waveguide length will ultimately impact the SBS gain too, and further reducing the waveguide losses will be helpful to maintain a long effective waveguide length.

In summary, we report the first observation of high‐frequency backward stimulated Brillouin scattering in suspended Z‐cut TFLN waveguides with a rich landscape of acoustic modes confined in TFLN waveguides between 8 and 20 GHz, spreading multiple microwave bands. Our key finding is the identification of a Brillouin shift of 16.8 GHz with a notable gain coefficient of 23.8 ${\left(Wm\right)}^{-1}$, a Brillouin response only accessible through the removal of the substrate to enable confinement of phonons with acoustic velocities exceeding those of the substrate material. For the first time, Brillouin microspectroscopy experiments are performed with TFLN waveguides, enabling us to confirm the existence and characterize the high‐frequency acoustic modes measured with our stimulated Brillouin scattering setup.

The Brillouin shifts demonstrated here cover new microwave bands, including the Ku‐band (12–18 GHz) and K‐band (18–26.5 GHz), and approach the millimeter‐wave regime, opening on‐chip filtering for satellite, 5G/6G, and radar systems at frequencies previously inaccessible to integrated Brillouin platforms. Combined with TFLN's native electro‐optic functionality, these high‐frequency Brillouin interactions enable capabilities currently confined to fiber‐based or hybrid systems onto a single chip, including Brillouin lasers, low‐phase‐noise microwave synthesis [4, 55], and Brillouin‐based filtering of individual comb lines [56, 57]. Together, these capabilities establish a route to integrated microwave photonic processors operating across the X‐, Ku‐, K‐, and emerging millimeter‐wave bands.

## 7 Materials and Methods

### 7.1 Mode Calculations

The individual polarization fraction $f_{i}$ for i=x,y,z of an acoustic displacement field $\vec{u}$ is defined as follows $f_{i}=p_{i}/\left(p_{x}+p_{y}+p_{z}\right)$ with

(8) $$p_{i}=\iint |{\vec{u}}_{i}{|}^{2}\;dxdy.$$

### 7.2 Stimulated Brillouin Scattering Experiment

The Brillouin gain spectrum was characterized using a double intensity‐modulated pump‐probe lock‐in amplifier measurement technique based on ref. [15] and as depicted in Figure 8. By conducting backward SBS measurements only, the pump and probe are injected from opposite sides into the chip. A DFB laser diode (Thorlabs DFB1550P) at 1552 nm was used as a pump laser and a tunable laser source (Toptica DLC pro with CTL) operating around 1552 nm was used as the probe signal. Both the pump and probe are intensity‐modulated with Mach–Zehnder modulators (MZM, JDSU 10G Mod) fed by sinusoidal signals at10 MHz and a slightly higher frequency of 10.075 MHz. The pump signal is amplified to 2 W, whereas the probe signal is amplified to 144 mW using erbium‐doped fiber amplifiers (EDFA, Amonics AEDFA‐33‐B‐FA and AEDFA‐PA‐35‐B‐FA, respectively). Polarization controllers are used to ensure TE mode excitation as lensed PM fibers, rotated accordingly, are used to couple into the waveguide. The probe is swept during the measurement for a pump‐probe detuning between 5 and 25 GHz. The transmitted probe is measured with a photo detector (PD, Optilab PD‐23‐C‐AC) and then processed by a lock‐in amplifier (LIA, Zurich Instruments), referenced at 75 kHz. The lock‐in amplifier is used to isolate and extract information about the SBS gain of both the fiber and the waveguide. For the measurements in this paper, we use a pump power of 30 dBm to ensure enough on‐chip power considering the facet losses of 7.7 dB.

**FIGURE 8:**
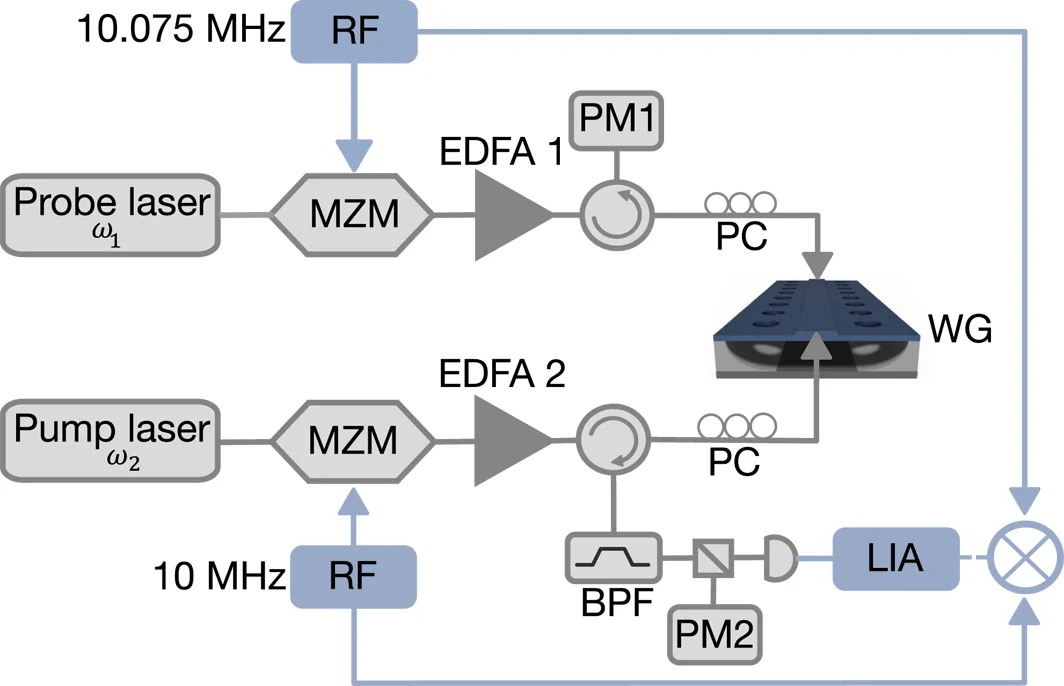
Schematic setup of the stimulated Brillouin scattering experiment. Pump‐probe experiment [15], BPF, bandpass filter; EDFA, erbium‐doped fiber amplifier; LIA, lock‐in amplifier; MZM, Mach–Zehnder modulator; PC, polarization controller; PD, photodetector; PM, power meter; RF, radio frequency.

The Brillouin gain coefficient $g_{B}$ is extracted by comparing the strength of the fiber SBS peak and the waveguide peaks in the measurement:

(9) $$g_{B,wg}=\frac{V_{\text{wg}}}{V_{\text{fibre}}}\frac{g_{\text{B,fibre}}P_{\text{pump,fiber}}L_{\text{eff,fiber}}}{P_{\text{pump,wg}}L_{\text{eff,wg}}}$$

where $V_{\mathrm{wg}}$ and $V_{\mathrm{fiber}}$ are the waveguide and fiber amplitude extracted from the LIA, respectively. $P_{\text{pump,wg}}$ and $P_{\text{pump,fiber}}$ are the pump power in the fiber and on‐chip, with $P_{\text{pump,wg}}$ being reduced by the facet reflections. $L_{\text{eff,fiber}}$ and $L_{\text{eff,wg}}$ are the effective lengths of fiber and waveguide, considering their propagation losses.

### 7.3 Brillouin Microspectroscopy

The Brillouin microspectroscopy setup consists of a continuous‐wave single‐frequency laser (660 nm, Torus, Laser Quantum, Novanta Photonics), a confocal microscope (CM1, Table Stable) and a 6‐pass scanning tandem Fabry–Pérot interferometer (TFP‐1, Table Stable) and is depicted in Figure 9a. We use a 20× microscope objective (Mitutoyo, NA = 0.42, WD = 20 mm) to focus light on the TFLN waveguide at the average optical power of 40 mW. Light scattered from the sample is collected in the backward direction by the same objective and directed to the TFP‐1 interferometer for spectral analysis. The elastically scattered (Rayleigh) light is suppressed using a mechanical shutter, leading to the instrument's sensitivity in the detection of Brillouin light scattering peaks on the order of $10^{10}$.

**FIGURE 9:**
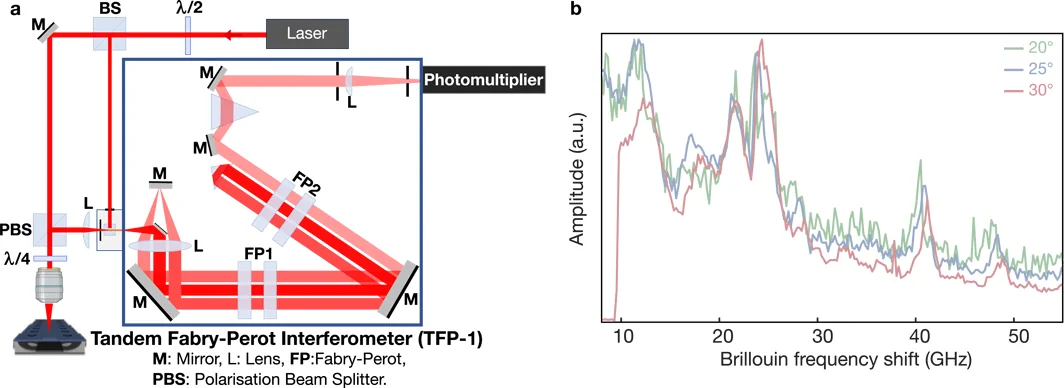
Spontaneous Brillouin light scattering experiment. (a) Schematic setup of Brillouin microspectroscopy; (b) spontaneous Brillouin spectra for various tilting angles θ=20°, 25° and 30°.

Brillouin spectra are acquired using the Ghost acquisition software (Table Stable). Each spectrum contains a Brillouin doublet (Stokes and anti‐Stokes peaks), symmetrically positioned around the laser frequency. The raw spectra are fitted using a Lorentzian line shape to determine each peak's central position, corresponding to the Brillouin frequency shift. The latter reflects the acoustic modal properties of the TFLN waveguide. Due to low efficiency of the spontaneous Brillouin scattering process, signals were acquired over extended time intervals (1–10 min) to improve the signal‐to‐noise ratio and the fitting precision. Multiple spectra were recorded at selected locations along the waveguide to ensure measurement repeatability. In addition, the waveguide was tilted between 20° to 30° relative to the optical axis of the objective lens to reduce stray light entering the spectrometer and to access scattering by phonons with a wavevector component parallel to the waveguide surface [42]. Three Brillouin gain spectra for θ=20°,25° and 30° are depicted in Figure 9b.

## Author Contributions

**Lisa‐Sophie Haerteis:** conceptualization, methodology, data curation, visualization, investigation, funding acquisition, writing – original draft, writing – review and editing, software, formal analysis. **Hadi Mahmodi:** data curation, methodology, writing – review and editing. **Yunlong Qiang:** writing – review and editing, visualization, software, formal analysis. **Yan Gao:** writing – review and editing, resources. **Madeline Hennessey:** writing – review and editing, data curation. **Peter Thurgood:** writing – review and editing, resources. **Jochen Schröder:** writing – review and editing, resources, supervision. **Arnan Mitchell:** supervision, resources, writing – review and editing. **Irina V. Kabakova:** supervision, writing – review and editing, methodology, conceptualization, resources, investigation. **Michael J. Steel:** writing – review and editing, writing – original draft, funding acquisition, supervision, conceptualization, methodology, investigation, formal analysis, software, project administration. **Andreas Boes:** writing – review and editing, writing – original draft, funding acquisition, project administration, supervision, conceptualization, resources, investigation, methodology.

## Funding

This work has been supported through the Australian Research Council (Grant Nos. DP220100488, DE230100964 and CE230100006) and the Swedish Research Council (Grant No. VR‐2021‐04241).

## Conflicts of Interest

The authors declare no conflicts of interest.

## Data Availability

The data that support the findings of this study are available from the corresponding author upon reasonable request.
